# Supplementary material for: Antenatal care service utilization disparities between urban and rural communities in Ethiopia: A negative binomial Poisson regression of 2019 Ethiopian Demography Health Survey
Source: PLoS One. 2024 Mar 14;19(3):e0300257. doi: 10.1371/journal.pone.0300257 (PMC10939242; doi:10.1371/journal.pone.0300257)
Supplement: S1 File — (DOCX) [file pone.0300257.s001.docx]

**STROBE Statement—checklist of** **Antenatal care service utilization disparities between urban and rural communities in Ethiopia: a negative binomial Poisson regression of 2019 Ethiopian Demography Health Survey study**

|  | Item No. | Recommendation | Page  No. | Relevant text from manuscript |
| --- | --- | --- | --- | --- |
| **Title and abstract** | 1 | (*a*) Indicate the study’s design with a commonly used term in the title or the abstract | 1 | Demography Health Survey |
|  |  | (*b*) Provide in the abstract an informative and balanced summary of what was done and what was found | 2 & 3 | The Ethiopian DHS of 2019 was used as a data source.  A negative binomial Poisson regression statistical model was used to analyse the data.  The number of antenatal care utilization is low among rural residents than urban residents. |
| Introduction | | | |  |
| Background/rationale | 2 | Explain the scientific background and rationale for the investigation being reported | 5 & 6 | Despite the inequalities in ANC service utilization among urban and rural women, there is limited evidence that shows the current disparities in ANC service utilization among this population group using national-level representative data that considers the count model after rural health extension programs have excelled in urban settings.  The finding of this study could lead to policy recommendations in order to improve maternal healthcare services in general. |
| Objectives | 3 | State specific objectives, including any prespecified hypotheses | 6 | The aim of this further DHS data analysis is to compare the level as well as the factors of antenatal care service utilization in rural and urban Ethiopia. |
| Methods | | | |  |
| Study design | 4 | Present key elements of study design early in the paper | 6 | The study was based on the intermediate EDHS 2019 dataset, which was conducted by the Central Statistical Agency in collaboration with the Federal Ministry of Health (FMoH) and the Ethiopian Public Health Institute. The survey was conducted from March 21, 2019 to June 28, 2019, based on a nationally representative sample (please check the 2019 EDHS report for more information) [28]. A cross-sectional study design using secondary data from 2019 EDHS was conducted. |
| Setting | 5 | Describe the setting, locations, and relevant dates, including periods of recruitment, exposure, follow-up, and data collection | 6 | The study was conducted in Ethiopia, located in north-eastern Africa. The study was based on the intermediate EDHS 2019 dataset, which was conducted by the Central Statistical Agency in collaboration with the Federal Ministry of Health (FMoH) and the Ethiopian Public Health Institute. The survey was conducted from March 21, 2019 to June 28, 2019, based on a nationally representative sample (please check the 2019 EDHS report for more information) [28]. |
| Participants | 6 | (*a*) *Cohort study*—Give the eligibility criteria, and the sources and methods of selection of participants. Describe methods of follow-up  *Case-control study*—Give the eligibility criteria, and the sources and methods of case ascertainment and control selection. Give the rationale for the choice of cases and controls  *Cross-sectional study*—Give the eligibility criteria, and the sources and methods of selection of participants | 7 | A nationally representative sample of 8,663 households provided 8,855 women of reproductive age (aged 15 to 49) as the source population for this study. The study population was 3,979 women who were in the reproductive age group (15–49 years) and have had pregnancy in the previous five years before the data collection period and living in Ethiopia. Hence, 3,962 (3,916.7 weighted) women data were extracted from the 2019 intermediate EDHS datasets. After excluding 17 women who had unknown number of ANC visits (missing data).  In light of this, the sample was stratified and selected in two steps. There were 21 sampling strata created in the first step after stratification was done by region and then by urban and rural areas within each region. In each sampling stratum, 305 enumeration areas (EAs) (94 urban and 211 rural) were chosen with a probability proportionate to the EA size. In the second stage, households were selected proportionally from each EA by using a systematic sampling method. Furthermore, the 2019 EMDHS provided details on the survey's design and methodology [28]. |
|  |  | (*b*) *Cohort study*—For matched studies, give matching criteria and number of exposed and unexposed  *Case-control study*—For matched studies, give matching criteria and the number of controls per case | NA |  |
| Variables | 7 | Clearly define all outcomes, exposures, predictors, potential confounders, and effect modifiers. Give diagnostic criteria, if applicable | 7 & 8 | The outcome variable of this study was the number of antenatal care (ANC) visits during last pregnancy between urban and rural women who were in the reproductive age group (15–49 years) and have had pregnancy in the previous five years before the data collection period. Both individual and household-level factors were assessed. The independent variables include; women’s age, religion, current marital status, educational level, household wealth index, family size and number of children “Table 1”. |
| Data sources/ measurement | 8* | For each variable of interest, give sources of data and details of methods of assessment (measurement). Describe comparability of assessment methods if there is more than one group | *8* | *Table 1. Description of individual and household level variables* |
| Bias | 9 | Describe any efforts to address potential sources of bias | 7 | The intermediate EDHS used a complete list of 149,093 enumeration areas (EAs) created for the upcoming Ethiopia population and housing census as a sampling frame. The frame comprises information about the EA type of residence (urban or rural), and estimated number of residential households.  Furthermore, the 2019 EMDHS comprised details on the survey's design and methodology. |
| Study size | 10 | Explain how the study size was arrived at | 7 | The intermediate EDHS used a complete list of 149,093 enumeration areas (EAs) created for the upcoming Ethiopia population and housing census as a sampling frame. The frame comprises information about the EA type of residence (urban or rural), and estimated number of residential households.  A nationally representative sample of 8,663 households provided 8,855 women of reproductive age (aged 15 to 49) as the source population for this study. The study population was 3,979 women who were in the reproductive age group (15–49 years) and have had pregnancy in the previous five years before the data collection period and living in Ethiopia. Hence, 3,962 (3,916.7 weighted) women data were extracted from the 2019 intermediate EDHS datasets. After excluding 17 women who had unknown number of ANC visits (missing data). |

Continued on next page

| Quantitative variables | 11 | Explain how quantitative variables were handled in the analyses. If applicable, describe which groupings were chosen and why | 8 | Table 1. Description of individual and household level variables |
| --- | --- | --- | --- | --- |
| Statistical methods | 12 | (*a*) Describe all statistical methods, including those used to control for confounding | 8 & 9 | Data cleaning was conducted to check for the consistency with the intermediate EDHS 2019 descriptive report. Recoding, variable generation, labelling and analysis were done by using STATA version 14.0. In the EDHS, the sample distribution between urban and rural settings was not proportionate. Therefore, sample weights were used to estimate frequencies to account for disproportionate sampling. The weighting procedure was meticulously explained in the 2019 EDHS report.  To handle over-dispersion of the data, we have considered Negative Binomial Poisson Model, the extension of Poisson regression to have precise result.  The analysis was done for both the urban and rural part. Finally incident rate ratio and odds ratio were presented with 95% CI. Statistical significance was declared at a p-value of less than 0.05. |
|  |  | (*b*) Describe any methods used to examine subgroups and interactions | 9 | Frequency, percentage, and mean were calculated for the explanatory and response variables using descriptive statistical analysis. Chi-square test was done to see if there was any association between ANC utilization among urban and rural residences and a statistically significant difference was observed between the two groups (χ2 = 437.51, p < 0.001), indicating that the factors associated with ANC utilization could be different among rural and urban residences. Therefore, the analysis was conducted separately. |
|  |  | (*c*) Explain how missing data were addressed | 7 | After excluding 17 women who had unknown number of ANC visits (missing data) |
|  |  | (*d*) *Cohort study*—If applicable, explain how loss to follow-up was addressed  *Case-control study*—If applicable, explain how matching of cases and controls was addressed  *Cross-sectional study*—If applicable, describe analytical methods taking account of sampling strategy | 7 | In the EDHS, the sample distribution between urban and rural settings was not proportionate. Therefore, sample weights were used to estimate frequencies to account for disproportionate sampling. The weighting procedure was meticulously explained in the 2019 EDHS report [24]. |
|  |  | (*e*) Describe any sensitivity analyses | NA |  |
| Results | | | | |
| Participants | 13* | (a) Report numbers of individuals at each stage of study—eg numbers potentially eligible, examined for eligibility, confirmed eligible, included in the study, completing follow-up, and analysed | 6&7 | A nationally representative sample of 8,663 households provided 8,855 women of reproductive age (aged 15 to 49) as the source population for this study. The study population was 3,979 women who were in the reproductive age group (15–49 years) and have had pregnancy in the previous five years before the data collection period and living in Ethiopia. Hence, 3,962 (3,916.7 weighted) women data were extracted from the 2019 intermediate EDHS datasets. After excluding 17 women who had unknown number of ANC visits (missing data). |
|  |  | (b) Give reasons for non-participation at each stage | 6 | 8,855 women of reproductive age (age 15-49).  3,979 women who were in the reproductive age group (15–49 years) and have had pregnancy in the previous five years before the data collection period.  After excluding 17 women who had unknown number of ANC visits (missing data).  3,962 (3,916.7 weighted) women data were extracted from the 2019 intermediate EDHS datasets. |
|  |  | (c) Consider use of a flow diagram | 6 | Fig 1: Study population and sampling procedures to identify the final sample size in 2019 EMDHS |
| Descriptive data | 14* | (a) Give characteristics of study participants (eg demographic, clinical, social) and information on exposures and potential confounders | 10 - 12 | The frequency of ANC visits was higher for pregnant women in age group 25-29 in both urban and rural pregnant women than in other age groups. One third 358 (36.09%) urban and 940 (31.65%) rural respondents had attained primary school. Utmost numbers of respondents 442 (44.56%) in urban and 1,410 (47.47%) in rural were Muslim religion followers. Based on the urban and rural segregated wealth index, most respondents 385(38.81%) in urban residence were richest; whereas 987(33.23%) of them were poorest. In both the urban 882 (88.91%) and rural 2,753(92.69%) residences married pregnant women holds the majority by current marital status “Table 3”. |
|  |  | (b) Indicate number of participants with missing data for each variable of interest | 7 | After excluding 17 women who had unknown number of ANC visits (missing data). |
|  |  | (c) *Cohort study*—Summarise follow-up time (eg, average and total amount) | NA |  |
| Outcome data | 15* | *Cohort study*—Report numbers of outcome events or summary measures over time | *NA* |  |
|  |  | *Case-control study—*Report numbers in each exposure category, or summary measures of exposure | *NA* |  |
|  |  | *Cross-sectional study—*Report numbers of outcome events or summary measures | *10 & 11* | *From 3962 (weighted 3916.67) pregnant women, about 155 (15.21%) urban and 848 (29.29%) rural residences of the pregnant women did not use antenatal care services in 2019, whereas 602 (59.10%), and 1085 (37.47%) of urban and rural pregnant women used four and more antenatal care services, respectively. The mean and variance of observations among urban residents were 3.69 and 4.89, and they are 2.59 and 4.22 among rural residents (Figs 2 and 3) and (Table 2).* |
| Main results | 16 | (*a*) Give unadjusted estimates and, if applicable, confounder-adjusted estimates and their precision (eg, 95% confidence interval). Make clear which confounders were adjusted for and why they were included | 14 - 16 | By keeping other variables constant, in the Negative Binomial Poisson Model, maternal age, educational status, and household wealth index variables become significant predictors for the low frequency of ANC service utilization among urban residences. Similarly, educational status, religion, household wealth index, and marital status show a significant association with the frequency of antenatal care service utilization among rural residences. |
|  |  | (*b*) Report category boundaries when continuous variables were categorized | 12 & 13 | Table 3: Sociodemographic characteristics disparity of antenatal care services utilization between urban and rural communities in Ethiopia, 2019 |
|  |  | (*c*) If relevant, consider translating estimates of relative risk into absolute risk for a meaningful time period | 14 - 16 | The use of ANC had increased with the level of education of women in both urban and rural residences. The number of antenatal care visits increased with 1.18 (IRR = 1.18, 95% CI: 1.07-1.30), 1.26 (IRR =1.26, 95% CI: 1.13-1.42), and 1.25 (IRR = 1.25, 95% CI: 1.11-1.41) times higher as the educational level increased with one unit among primary, secondary, and higher educated women than no education women in urban residences, respectively. Whereas, as the educational level increases with one unit, antenatal care visits increases by 1.34 (IRR = 1.34, 95% CI: 1.24-1.45), 1.54 (IRR =1.54, 95% CI: 1.34-1.76), and 1.58 (IRR = 1.58, 95% CI: 1.28-1.95) times higher among primary, secondary, and higher educated women than no education women in rural residences, respectively. |

Continued on next page

| Other analyses | 17 | Report other analyses done—eg analyses of subgroups and interactions, and sensitivity analyses | 8 & 14 | Chi-square test was done to see if there was any association between ANC utilization among urban and rural residences and a statistically significant difference was observed between the two groups (χ2 = 437.51, p < 0.001), indicating that the factors associated with ANC utilization could be different among rural and urban residences. Therefore, the analysis was conducted separately.  The estimates of the log likelihoods of both AIC and BIC slightly support the adoption of the NB model in both the urban and rural analyses. Of the two different models (Poisson and NB) being fitted, NB has the lowest AIC (4300.28) and BIC (4415.98) in both final models (Table 4). |
| --- | --- | --- | --- | --- |
| Discussion | | | | |
| Key results | 18 | Summarise key results with reference to study objectives | 18 | The study revealed that the mean antenatal care visits and utilization of pregnant women in urban residences were higher than in rural residences. In this study, pregnant women’s educational level was an important factor that determined ANC utilization regardless of the type of residence. |
| Limitations | 19 | Discuss limitations of the study, taking into account sources of potential bias or imprecision. Discuss both direction and magnitude of any potential bias | 20 | The study also has some limitations. Showing temporal relationship between ANC service utilization and its predictors was impossible due to the type of the study design, cross-sectional, used for the survey. |
| Interpretation | 20 | Give a cautious overall interpretation of results considering objectives, limitations, multiplicity of analyses, results from similar studies, and other relevant evidence | 17 - 18 | The study revealed that the mean antenatal care visits and utilization of pregnant women in urban residences were higher than in rural residences. This finding is in line with the study by Enyew and Mekonnen [[30](#_ENREF_30)], who reported that women living in urban areas had a higher expected number of antenatal care visits during their pregnancy than women living in rural areas. According to this finding, there was a significant association between ANC utilization and age group. Age groups of pregnant women 20-24, 25-29, 30-34, 35-39, 40-45 were more likely to utilize ANC than the 15-19 age group of pregnant women in urban residences. This was consistent with the previous study in Ethiopia [30], which found older pregnant mothers (in the age range of 35 to 49 years) in 2011 used ANC more frequently. It is also in line with the study conducted in Tanzania [32] and Rwanda [33], which showed that pregnant women between the ages of 15-19 years were more likely to use antenatal care services than those over the age of 19. Furthermore, this finding was inconsistent with a study conducted in Nepal [34], which revealed that women over the age of 35 were less likely to seek prenatal care, and a prior study [22], which showed no connection between antenatal care service use in Ethiopia and younger age. |
| Generalisability | 21 | Discuss the generalisability (external validity) of the study results | 20 | However, the researchers believe that the aforementioned limitations cannot significantly impair the validity of the study's conclusions. |
| Other information | |  | | |
| Funding | 22 | Give the source of funding and the role of the funders for the present study and, if applicable, for the original study on which the present article is based | NA |  |

*Give information separately for cases and controls in case-control studies and, if applicable, for exposed and unexposed groups in cohort and cross-sectional studies.

**Note:** An Explanation and Elaboration article discusses each checklist item and gives methodological background and published examples of transparent reporting. The STROBE checklist is best used in conjunction with this article (freely available on the Web sites of PLoS Medicine at http://www.plosmedicine.org/, Annals of Internal Medicine at http://www.annals.org/, and Epidemiology at http://www.epidem.com/). Information on the STROBE Initiative is available at www.strobe-statement.org.
