# Supplementary material for: Antenatal care service utilization disparities between urban and rural communities in Ethiopia: A negative binomial Poisson regression of 2019 Ethiopian Demography Health Survey
Source: PLoS One. 2024 Mar 14;19(3):e0300257. doi: 10.1371/journal.pone.0300257 (PMC10939242; doi:10.1371/journal.pone.0300257)
Supplement: Attachment — Submitted filename: ANC_Ethiopia_Count Model_Review.docx [file pone.0300257.s003.docx]

**Abstract**

The abstract extends far beyond the word limit required by the journal which should be about 300 words and I feel that the authors need to abide by this word limit.

The background section of the abstract does not seem to present any rationale for this study which should inform the objective of the study. This is because obviously, there are numerous studies in the context of Ethiopia that have explored ANC utilization and geographical disparities inherent in it. Providing the justification for the study here in the background will give the readers a quick idea of why the study is important and needed.

The result section presented in the abstract will need to be written again to present the most important and significant result, which will ensure that the authors align with the 300-word limit required by the journal.

The conclusion section of the abstract seems like a summary of the results already presented in the previous section. The authors should provide a better conclusion for their study and provide policy or programmatic recommendations or implications for future studies in Ethiopia.

**Introduction**

The paper will require professional editing services to rid it of various grammatical issues.

Lines 73-76 can be added to the previous paragraph.

The information in lines 83-88 should be added to lines 67-72 because the information being discussed in both sections is similar and falls under the same theme of ANC recommendations and utilization.

Most earlier studies talked about in lines 77-78, what have they found in urban areas? Is the aim of this study to compare evidence from rural areas with these studies??

Has there been no study in Ethiopia where the level of ANC in rural and urban areas has been compared? I am afraid that you have not provided enough justification for your study and this needs to be clearly provided in this study. Also, the gap could be methodological, if previous studies have not employed count models to study ANC, you can fill that gap in addition to what you have discussed as the justification for the present study.

**Methodology**

To avoid going beyond the journal word limit, I think there isn’t any need to provide much information on the study setting. Rather, those words can be used to provide more information about the EDHS data (check the 2019 EDHS report for more information).

On what basis were the study variables (independent variables) selected for this study? Did the authors consider the need to include variables are the household and community level considering that the study aims to compare urban and rural contexts?

The authors need to provide more information on the outcome variable. How was the question asked in the survey? What did the response from the respondents look like? Is the response a count variable??

While it is understandable that your data is over-dispersed and violates the assumptions of Poisson regression, hence the need for other count models. I think it will be good to better inform your readers about the reason behind your decision to choose negative binomial regression. What does the distribution of your outcome variable look like? Is the outcome variable zero-inflated? These and more are issues to discuss in order for NB regression to be considered the most suitable.

Also, the authors should provide more information on model selection criteria.

The authors can also provide a bar chart showing the distribution of the outcome variable [ANC].

It would also be great to see the authors present mathematical notations of their NB regression models (see previous studies that have employed NB regression for this).

Similar studies that have employed count models to study ANC that could provide guidance to the authors

<https://link.springer.com/article/10.1007/s40745-021-00328-x>

<https://www.tandfonline.com/doi/full/10.1080/03630242.2016.1222325>

<https://journals.sagepub.com/doi/full/10.1177/24551333211030349>

<https://journals.plos.org/plosone/article?id=10.1371/journal.pone.0228215>

**Results**

The authors should consult some other studies that have employed count models for the best practices in the interpretation of the results of count models, especially the IRR.
